# Sleepless Longing: Bidirectional Associations Between Sleep Quality and Prolonged Grief in Daily Life After Traumatic Loss

**DOI:** 10.1002/cpp.70238

**Published:** 2026-02-12

**Authors:** L. M. M. Kivelä, J. Pociūnaitė‐Ott, L. I. M. Lenferink

**Affiliations:** ^1^ Department of Psychology, Health and Technology, Faculty of Behavioural Management and Social Sciences University of Twente Enschede the Netherlands

**Keywords:** bereavement, ecological momentary assessment, experience sampling method, insomnia, sleep diary

## Abstract

Sleep disturbances are common in bereavement, especially among those with prolonged grief disorder (PGD). While cross‐sectional and longitudinal studies have linked PGD with poor sleep, the directionality and timing of these effects remain unclear. In the present study, we employed a 14‐day ecological momentary assessment (EMA) protocol to examine the day‐to‐day associations between sleep and grief in a sample of 46 traumatically bereaved adults (*M*_age_ = 56.0, 78% female, *Med*_time since loss_ = 3.8 years). PG symptoms were assessed 5×/day and sleep quality each morning via a smartphone app. Linear mixed models were used to examine within‐person, lagged effects of sleep quality on PG levels and vice versa. Results indicated that PG symptoms did not predict sleep quality the following night. However, lower sleep quality was associated with increased PG severity during the day (controlling for previous‐day PG levels), although this effect was no longer significant in sensitivity analyses restricted to participants with more complete sleep data (≥ 50%). Exploratory post hoc analyses indicated that sleep quality was most strongly associated with PG levels in the morning, with the effect being attenuated later in the day. Altogether, these findings provide tentative support for a night‐to‐day, sleep‐to‐grief pathway, although both small effect sizes and variability in results underscore the need for replication in larger samples. While preliminary, these findings suggest that poor sleep may contribute to the exacerbation of PG symptoms in the short term, highlighting the need for further research into the temporal dynamics and potential causal mechanisms linking sleep and grief.

## Summary

- Sleep problems in traumatically bereaved adults are linked to increases in grief intensity in daily life.
- Grief intensity, in turn, does not appear to negatively impact sleep.
- Morning hours may represent a particularly vulnerable period following disturbed sleep.
- Sleep represents a potentially modifiable treatment target in the prevention and treatment of prolonged grief disorder.

## 1 Introduction

Sleep disturbances commonly occur after losing a loved one and may range from difficulties initiating or maintaining sleep (i.e., early or middle insomnia) to poor sleep quality and nightmares (Lancel et al. 2020; Monk et al. 2008). Among those with prolonged grief disorder (PGD), these problems are even more prevalent, with up to 80% of those with PGD reporting clinically relevant sleep complaints (Germain et al. 2005). PGD is characterized by intense longing for, or preoccupation with, the deceased and intense emotional pain (e.g., sadness) that continue to cause significant functional impairment more than 6 months (in ICD‐11) (World Health Organization 2022) or 12 months (in DSM‐5‐TR) (American Psychiatric Association 2022) after the loss. PGD affects roughly 3%–5% of bereaved individuals (Rosner et al. 2021; Treml et al. 2024), although prevalence rates are about five times higher among those bereaved by a sudden or violent death, such as a homicide, suicide or accident (Djelantik et al. 2020).

Despite the frequent co‐occurrence of PG symptoms and sleep disturbances, the directionality of this relationship remains unclear. Some studies suggest that sleep complaints may precede and pose a risk factor for PGD. For instance, in a cross‐lagged panel study of 343 bereaved adults, insomnia symptoms at baseline predicted PG intensity at both 6‐ and 12‐month follow‐up, but not vice versa (de Lang et al. 2023). Yet, in a follow‐up analysis of the same dataset, the reverse association was also found: Baseline PG intensity predicted the persistence of sleep complaints over time. Moreover, the symptom trajectories of insomnia and PG symptoms were highly correlated, based on a parallel process model of changes in insomnia and PG symptoms over time (de Lang et al. 2024). Together, these findings suggest that sleep and grief may form a reciprocal, mutually reinforcing cycle, rather than exhibiting simple unidirectional effects.

Other longitudinal studies support this possibility, showing that PG symptoms predict poor sleep outcomes over time (see, e.g., Lancel et al. 2020). However, the opposite direction (i.e., sleep predicting PG symptoms) has been less frequently explored. Further, most of this work has relied on traditional, longitudinal study designs where assessments are spaced months or years apart. However, these methods are poorly suited to capturing the day‐to‐day fluctuations and temporal dynamics that characterize both sleep and PG symptoms. PG levels are known to vary considerably in daily life (L. I. M. Lenferink et al. 2024), and sleep quality is similarly sensitive to fluctuations across days (Bei et al. 2016). As such, designs that average across long time periods may obscure key insights into how these bidirectional associations between sleep and PG symptoms unfold in real time.

Ecological momentary assessment (EMA) provides a valuable methodological alternative. By repeatedly sampling individuals in their natural environments, EMA allows for a fine‐grained analysis of symptom variability and temporal ordering with increased ecological validity (Shiffman et al. 2008). While prior EMA studies on sleep and PG symptoms are lacking, there is reason to expect that sleep and PG symptoms are bidirectionally associated in daily life. Prior research using daily sleep diaries and EMA to examine fluctuations in affect has shown that sleep and negative affect influence each other across days: Poor sleep predicts lower mood and emotional reactivity the following day and vice versa (Hickman et al. 2024; Triantafillou et al. 2019). Theoretical models of sleep–affect interactions also suggest that emotion dysregulation (which may present, for example, as preoccupation with thoughts) can disrupt restorative sleep, while poor sleep in turn impairs one's ability to regulate their emotions the following day (Palmer and Alfano 2017). Accordingly, poor sleep has also been shown to worsen specific cognitive‐affective states (such as hopelessness) on a night‐to‐day basis (Kivelä et al. 2024). Further, cognitive processes implicated in PGD (such as preoccupation with thoughts) are all well‐established risk factors for insomnia (Harvey 2002; Lamprou et al. 2024; Pillai et al. 2014).

Applied to bereaved people, research on sleep–affect dynamics would therefore suggest that heightened PG symptoms may contribute to poorer sleep at night and that disturbed sleep in turn may exacerbate PG symptoms during the day, therefore forming a potential feedback loop. The present study therefore aimed to examine the night‐to‐day, within‐person associations between sleep quality and PG symptoms using EMA data, which was collected over 14 days in a sample of traumatically bereaved individuals.

## 2 Methods

### 2.1 Study Design

An intensive longitudinal cohort study. This study was preregistered prior to data analysis (osf.io/zrym3).

### 2.2 Sample

Data were drawn from the *Grief‐ID Archive* (L. Lenferink and Pociūnaitė‐Ott 2025; Pociūnaitė‐Ott and Lenferink 2025), an archive dataset of EMA studies focused on grief in daily life. For the present study, we used data from a project including 70 traumatically bereaved adults (i.e., individuals who had lost a loved one due to suicide, homicide, or accident at least 12 months prior). Participants were recruited via an online screener on the website www.rouwbehandeling.nl (‘Grief Treatment’), a psychoeducational platform for bereaved individuals. Further inclusion criteria included reporting elevated levels of PG (i.e., above the clinical cut‐off ≥ 71; L. I. M. Lenferink, Eisma, et al. 2022) at the time of completing the online screener. For the present analyses, a subset of *N* = 46 participants who provided ≥ 30% of daily sleep data were retained.

### 2.3 Procedure

At baseline, participants completed questionnaire measures assessing sociodemographics, loss characteristics and PG and related symptoms via an online survey platform (Qualtrics). Participants then commenced a 14‐day EMA protocol, during which they received five daily surveys via a mobile app (Avicenna) at semi‐random intervals (between 8:30–9:30, 11:30–12:30, 14:30–15:30, 17:30–18:30 and 20:30–21:30). After each prompt, participants had 60 min to complete the survey before it expired and received reminders 10 and 20 min after the initial prompt in case they had not yet completed the survey. Instructions for downloading and using the Avicenna app were provided through a video tutorial. More details on the study procedure can be found elsewhere (L. Lenferink and Pociūnaitė‐Ott 2025; Pociūnaitė‐Ott and Lenferink 2025; Specker et al. 2025).

### 2.4 Instruments

#### 2.4.1 Sociodemographic and Loss‐Related Characteristics and Psychopathology Symptoms at Baseline

Sociodemographics (age, gender, level of education) and loss‐related characteristics (time since loss, relation to the deceased, cause of death and whether participants were currently receiving help from a mental health professional regarding the death of their loved one) were assessed via a custom survey. Psychopathology symptom levels were examined using standardized self‐report measures assessing PG, post‐traumatic stress disorder (PTSD), depression symptoms and functional impairment. PG symptoms were assessed with the Traumatic Grief Inventory–Self Report Plus (TGI‐SR+) (L. I. M. Lenferink, Eisma, et al. 2022), with scores ≥ 71 indicating probable PGD. PTSD symptoms were measured using the PTSD Checklist for DSM‐5 (PCL‐5) (Blevins et al. 2015), with items worded specifically to reflect symptoms related to the death of a loved one; scores ≥ 31 on the PCL‐5 indicate probable PTSD (Bovin et al. 2016). Depression symptom severity was assessed using the Patient Health Questionnaire‐9 (PHQ‐9) (Kroenke et al. 2001), with scores ≥ 10 indicating probable major depression. Functional impairment was assessed using the Work and Social Adjustment Scale (WSAS) (Mundt et al. 2002), where scores ≥ 20 indicate moderately severe impairment.

#### 2.4.2 EMA of Sleep Quality and PG Symptoms

The present study focused on two variables as measured with EMA: daily sleep quality and daily PG intensity. PG symptoms were assessed at each beep with the two‐item Prolonged Grief Symptoms–Short Ecological Assessment (PGS‐SEA) (Ergun et al. 2025) scale: ‘In the past 3 hours, I found myself yearning for him/her’ and ‘In the past 3 hours, I felt sad because of his/her death’. Daily PG intensity was computed by averaging these two items across the five prompts per day. The two‐item EMA scale exhibits good psychometric properties, with a correlation of *r* = 0.72 with the TGI‐SR+ self‐report scale (Ergun et al. 2025). In the present sample, the intraclass correlation (ICC) for the two‐item scale was 0.67, indicating that 67% of the variance in the scores could be attributed to between‐person differences and 33% to within‐person variability. When the TGI‐SEA scores were averaged per day, the ICC value (0.79) indicated that 79% of the variance in the daily scores was attributable to between‐person and 21% to within‐person variability.

Subjective sleep quality was assessed with one item during the first beep of the day: ‘I slept well last night’ (Brüdern et al. 2022; Forkmann et al. 2018). In the present sample, the ICC for the sleep item was 0.36, indicating that 36% of the variance in the scores could be attributed to between‐person differences and 64% to within‐person variability. All EMA items were assessed on a 7‐point Likert‐type scale where 0 = *Not at all* and 6 = *Very much*.

### 2.5 Statistical Analysis

All analyses were conducted in R using the *lme4* package (Bates et al. 2015). Prior to analysis, all variables were person‐mean centred in order to estimate within‐person effects (Nezlek 2012). Two lagged linear mixed models (LMMs) were then specified, examining the association between PG symptoms during the day (*t*) and sleep quality the following night (*t* + 1), controlling for prior sleep quality (*t*) (Model 1), and the associations between sleep quality the previous night (*t*) and PG symptoms the following day (*t* + 1), controlling for PG levels the previous day (*t*) (Model 2).^1^ Both models included random intercepts and random slopes, with observations nested within individuals (i.e., specifying a two‐level structure). Hypothesis testing was based on within‐person effects, with *p* < 0.05 (two‐sided). Model fit was assessed via −2 log‐likelihood, Akaike information criterion (AIC) and Bayesian information criterion (BIC) and marginal/conditional *R* ^2^ values (indicating the variance explained by fixed effects only vs. the model as a whole; Nakagawa and Schielzeth 2013). In case of poor fit, models were simplified in accordance with steps outlined in the study preregistration (osf.io/zrym3). Other analyses included examining between‐person associations (i.e., the association between average sleep quality and PG levels) using simple linear regression. Sensitivity analyses included running the above models only among a subsample of participants (*n* = 38) who had ≥ 50% of the sleep data available.

A post hoc power calculation indicated that with *N* = 46 and *k* = 10 observations per participant on average, we had power (0.95) to detect medium effects (0.20) in a random intercept model. In a model incorporating both random intercepts and random slopes, we had power (0.90) to detect medium effects (0.20). These findings are consistent with power recommendations for multilevel models (Bell et al. 2014; Maas and Hox 2005).

## 3 Results

### 3.1 Sample Characteristics

Sociodemographic, loss‐related characteristics and psychopathology symptoms of the sample (*N* = 46) can be found in Table 1. The sample was predominantly female (78%), with a mean age of 56.0 years (SD = 10.0, range = 32–76). The mean duration since loss was 3.8 years (SD = 9.4, range = 1.1–47.6).

**TABLE 1:** Sample characteristics (*N* = 46).

Variable	Statistic	Range
Gender, *N* (%)		
Male	10 (22%)	—
Female	36 (78%)	—
Age, *M* (SD)	56.0 (10.0)	32–76
Level of education, *N* (%)		
Secondary school	5 (11%)	—
Vocational education	13 (28%)	—
Higher professional education/university	28 (61%)	—
Kinship to deceased, *N* (%)		
Partner	11 (24%)	—
Child	20 (43%)	—
Other	15 (33%)	—
Cause of death, *N* (%)		
Accident	18 (39%)	—
Suicide	26 (57%)	—
Murder/manslaughter	2 (4%)	—
Time since loss (years), *Med* (SD)	3.8 (9.4)	1.1–47.6
Currently receiving professional bereavement care, *N* (%)	10 (22%)	—
PG symptoms (TGI‐SR+), *M* (SD)	69.04 (12.93)	38–97
PTSD symptoms (PCL‐5), *M* (SD)	29.93 (12.74)	7–62
Depression symptoms (PHQ‐9), *M* (SD)	10.35 (5.31)	0–23
Functional impairment (WSAS), *M* (SD)	23.07 (9.93)	3–38
Average daily PG intensity, *M* (SD)	2.50 (1.67)	0–6
Average daily sleep quality, *M* (SD)	3.50 (1.65)	0–6

Abbreviations: PCL‐5 = PTSD Checklist for DSM‐5, PHQ‐9 = Patient Health Questionnaire‐9, TGI‐SR+ = Traumatic Grief Inventory–Self Report Plus, WSAS = Work and Social Adjustment Scale.

Participants overall completed on average 66% (*M* = 46, range 15–68) of the EMA, with data on PG symptoms being available for 93% (*M* = 13, range = 6–14) of the days per participant on average. The mean number of EMA available per person per day used to calculate the aggregate daily score was 4 (range = 2–5). Sleep data were available for 71% of the nights, with an average of *M* = 10 nights per person (range = 4–14).

### 3.2 Within‐Person Effects

Based on a model including both random intercepts and slopes, PG symptoms did not predict sleep quality the subsequent night (*B* = 0.10, SE = 0.13, *p* = 0.427). No significant autocorrelations for sleep quality were found (*B* = 0.03, SE = 0.06, *p* = 0.602) (Model 1; Table 2).

**TABLE 2:** Linear mixed‐models examining bidirectional associations between sleep quality and grief.

Model	*B* (SE)	CI 95%	*p*	AIC	BIC	−2 log‐likelihood	Marginal *R* ^2^	Conditional *R* ^2^
Model 1 (grief > sleep)				1284.81	1311.73	1270.81	0.002	0.389
Intercept	3.50 (0.18)	3.15; 3.85	< 0.001					
Grief	0.10 (0.13)	−0.15; 0.35	0.427					
Sleep (*t* − 1)	0.03 (0.06)	−0.08; 0.14	0.602					
Model 2 (sleep > grief)				1087.30	1115.87	1073.30	0.006	0.835
Intercept	2.47 (0.22)	2.03; 2.91	< 0.001					
Sleep	−0.05 (0.03)	−0.11; −0.01	0.045					
Grief (*t* − 1)	0.13 (0.04)	0.05; 0.21	0.001					

*Note:* All models include within‐person centred predictors and an uncentred outcome.

Examining the opposite direction of causality (i.e., sleep impacting PG severity, i.e., Model 2), we found a significant within‐person effect of sleep quality of PG levels during the day (*B* = −0.05, SE = 0.03, *p* = 0.045). This means that when people reported poorer sleep quality than usual, this was associated with higher than usual PG levels the next day. This model also found significant autocorrelations between PG levels across days (*B* = 0.13, SE = 0.04 *p* = 0.001).

**FIGURE 1:**
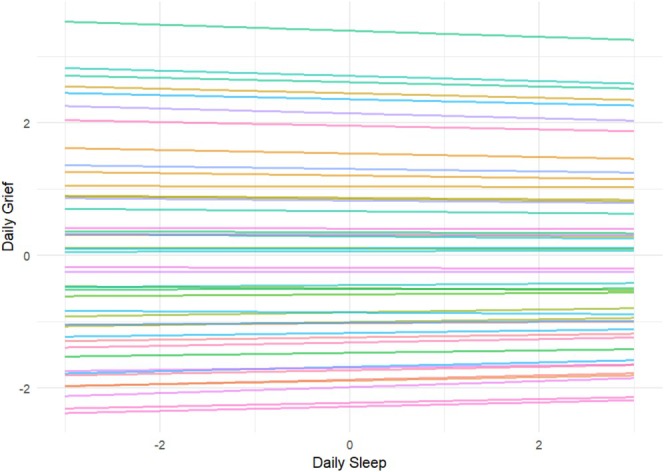
Person‐specific slopes of sleep quality on grief. *Note:* The x‐axis is centred at zero due to person‐mean centring of the predictor. The y‐axis reflects predicted grief based on person‐specific intercepts and slopes; values are relative to the sample mean.

Visual inspection of the person‐specific slopes (Figure 1) indicated an overall small, negative effect of sleep quality on PG levels. Considerable between‐person variability was observed in baseline PG levels (i.e., intercepts), but more limited variation was apparent in slopes. Most individual associations were relatively flat, indicating a modest effect of sleep on PG levels.

Model comparisons further supported the unidirectional nature of the effects, with Model 2 examining the effects of sleep on PG levels showing notably better fit (i.e., lower AIC/BIC values and a higher conditional *R* ^2^) than the opposite Model 1. However, marginal *R* ^2^ values remained low in both models, indicating that most of the variance in the outcomes was still accounted for by between‐person differences, rather than within‐person fluctuations (Nakagawa and Schielzeth 2013).

### 3.3 Post Hoc Analyses

In order to examine whether the effect of sleep quality on PG levels differed across the day, we conducted exploratory post hoc analyses^2^ using a LMM. The model included prior night's sleep quality, time of day (i.e., beep number from 1 to 5; dummy‐coded with beep 1 as the reference category) and their interaction (representing variation in the sleep–PG association between time points) as predictors of momentary PG levels, controlling for PG levels at the previous time point. There was a significant main effect of sleep quality (*B* = −0.13, SE = 0.04, *p* = 0.003), whereby poorer sleep predicted higher PG levels the following day. Notably, the coefficient for sleep quality increased in magnitude compared to the earlier model without time of day covariates (*B* = −0.05), suggesting that accounting for time‐of‐day variation strengthened the observed association between sleep and PG levels.

The main effect of time of day was not significant for most beeps; only beep 3 showed a significant negative effect (*B* = −0.23, SE = 0.08, *p* = 0.006), indicating that PG levels were lower in the middle part of the day (between 14:30 and 15:30); all other time points were non‐significant (all *p*s > 0.10).

**FIGURE 2:**
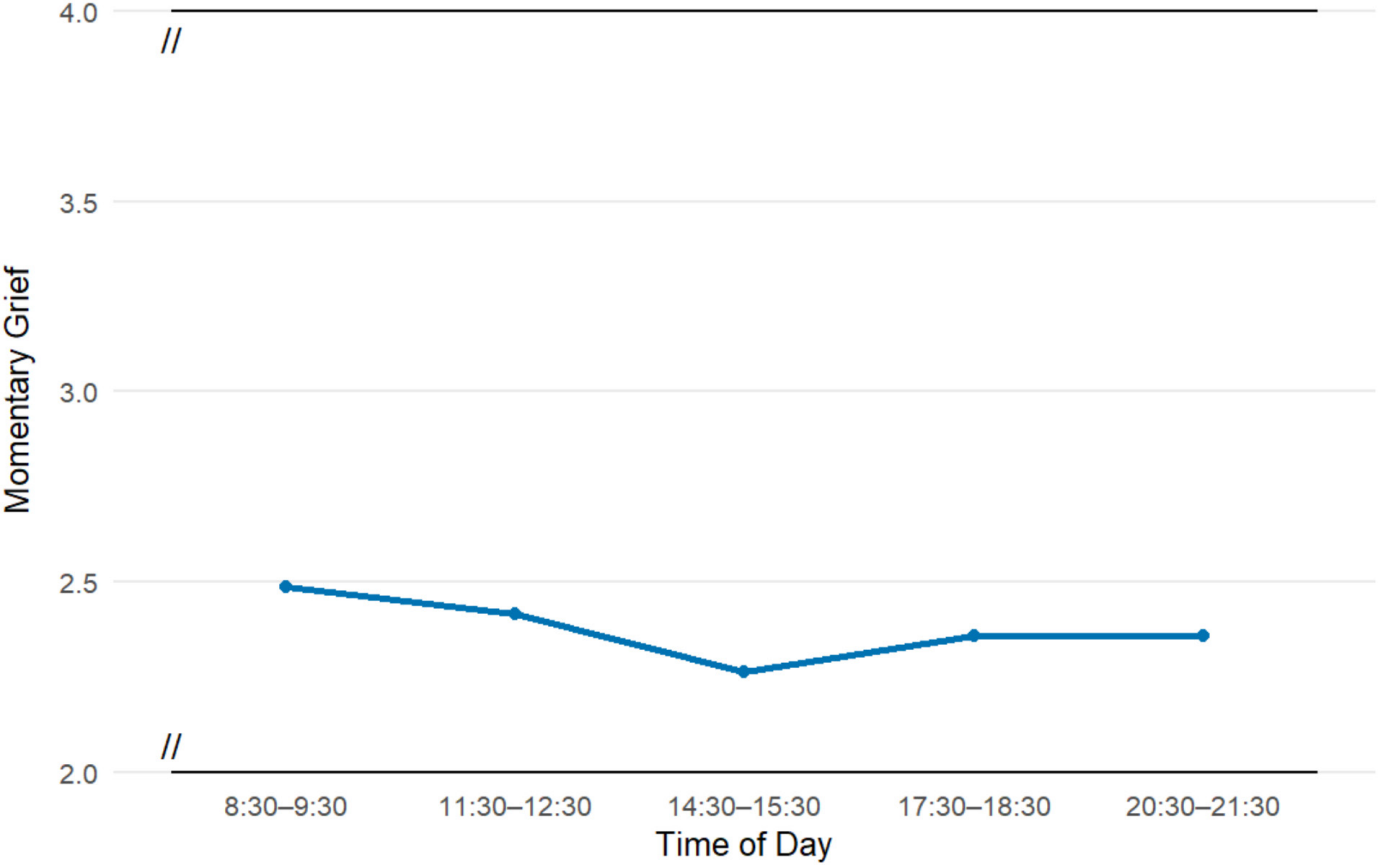
Within‐day variation in momentary grief (accounting for sleep quality). *Note:* y‐axis is truncated.

Significant interactions were found between sleep quality and time of day for beeps 2 (*B* = 0.18, SE = 0.06, *p* = 0.002) and 3 (*B* = 0.16, SE = 0.06, *p* = 0.004), indicating that the negative association between sleep and PG levels observed at beep 1 was attenuated later in the day. This suggests that sleep quality may be most strongly associated with grief in the earlier part of the day (see Figure 2 and Table S1 for full results).

### 3.4 Between‐Person Effects

When examining between‐person differences in average sleep quality and PG levels across the entire study period, we did not find a significant association between sleep quality and PG intensity, *F*(1, 61) = 1.32, *p =* 0.256. This indicates that individuals who, on average, reported poorer sleep did not experience higher levels of PG compared to those who reported better sleep.

### 3.5 Sensitivity Analyses

All main analyses were repeated in the subsample of participants who had ≥ 50% of the sleep data available, resulting in a sample of *n* = 38 with *M* = 11 nights of data available on average. In the sensitivity analyses, PG levels likewise did not emerge as a significant within‐person predictor of sleep quality. Further, the effect of sleep quality on PG became non‐significant, although the effect size for sleep remained unchanged (*B* = −0.05, SE = −0.03, *p* = 0.062) (see Table S2 for full results). A power calculation indicated that the sensitivity analyses were powered (0.90) to detect medium effects (0.20).

## 4 Discussion

The present study represents the first examination of bidirectional, within‐person associations between sleep quality and PG levels in daily life using EMA. Contrary to our expectations of bidirectional influences, we found no evidence that heightened PG levels during the day negatively affected sleep quality the subsequent night. On the contrary, lower sleep quality predicted increased PG levels during the day, although this effect was no longer significant in sensitivity analyses. Exploratory post hoc analyses indicated that sleep quality was most strongly associated with PG levels in the morning, with the effect being attenuated later in the day. Taken altogether, our findings offer tentative support for the notion that sleep quality predicts PG to a greater extent than vice versa.

The finding that sleep quality predicted next‐day PG levels aligns with previous EMA and sleep diary research showing that poor sleep impairs adaptive emotion regulation and increases negative affective states (Hickman et al. 2024; Triantafillou et al. 2019). Cognitive‐behavioural models of insomnia similarly posit that sleep disturbances can undermine an individual's ability to regulate distressing thoughts and emotions (Harvey 2002; Palmer and Alfano 2017). Based on our findings, it appears that this dysregulation may also extend to PG symptoms, such as yearning or sadness. This aligns with prior work indicating that poor sleep seems to increase hopelessness (Kivelä et al. 2024), with hopelessness/meaninglessness being a common by‐product of (prolonged) grief (Pociūnaitė‐Ott et al. 2026).

Notably, all of our participants were traumatically bereaved, which is known to be associated with an increased prevalence and severity of sleep disturbances (Germain et al. 2005). However, their loss had occurred more than 12 months prior (average time since loss 3.8 years), meaning that some sleep–grief effects (or vice versa) may have already become more nuanced over time (i.e., it is possible that sleep may be even more disturbed in the immediate aftermath of the loss, hence exhibiting tighter links with grief in the early weeks after bereavement). However, as the sleep–PG link did not retain significance in the sensitivity analyses, our results should be interpreted with caution, also due to the small sample size. These inconsistencies are likely to reflect the small magnitude and substantial between‐person variability in the effect, as suggested by the distribution of the person‐specific slopes (Figure 1).

In exploratory post hoc analyses, we further found that the association between sleep quality and PG levels varied across the day: The impact of poor sleep was strongest in the morning and appeared to diminish over the course of the day. This pattern aligns with the broader literature on diurnal (i.e., within day) variations in emotional functioning, as the early morning time appears to represent a period of heightened vulnerability (Balter et al. 2024; Lo et al. 2016; Murray et al. 2009). Sleep inertia, which refers to the residual fatigue experienced after waking up, is associated with reduced cognitive functioning, including emotion regulation, and increased negative affect (Tassi and Muzet 2000). This may render individuals more emotionally vulnerable in the morning hours, especially following a bad night's sleep. Later in the day, a recovery pattern is observed instead, with both mood and emotional functioning improving (Balter et al. 2024). While diurnal mood variation (specifically, early morning mood worsening) is also a classic symptom of many mental health disorders, such as depression (Balter et al. 2024; Kivelä et al. 2018; Morris et al. 2009), we are the first to demonstrate a similar pattern in PG intensity.

Another notable finding was that we did not find between‐person associations between sleep quality and PG levels, suggesting that these associations are not driven by stable between‐person differences, but rather reflect within‐person, day‐to‐day fluctuations in sleep that have the potential to impact PG levels on a next‐day basis. Interestingly, while we observed significant autocorrelations for PG levels (indicating some temporal stability in daily grief), sleep quality did not exhibit similar characteristics. This pattern suggests that sleep quality appears more susceptible to short‐term contextual or situational influences than grief, at least on the day‐level (although substantial within‐day fluctuations are observed; L. I. M. Lenferink et al. 2024). This finding aligns with prior studies showing considerable night‐to‐night variability in subjective sleep reports, particularly in highly‐stressed samples (Bei et al. 2016).

In contrast, we did not find evidence that elevated daytime PG symptoms impacted subsequent sleep quality. This is contrary to a previous longitudinal examination showing reciprocal associations between PG symptoms and sleep complaints, using three measurements over 12 months (de Lang et al. 2023, 2024). However, noteworthy differences in comparison to the present study include that prior longitudinal research relied only on recalling PG levels and sleep quality in the past 2–4 weeks, and data were collected months apart. Traditional longitudinal study designs may capture different dynamics than the night‐to‐day effects observed in our study. For example, grief‐induced sleep disturbances may accumulate more gradually, rather than being influenced by short‐term fluctuations. Finally, It is also possible that specific aspects of grief (e.g., preoccupation with thoughts; L. I. M. Lenferink, van Eersel, and Franzen 2022) are more likely to disrupt sleep; however, these were not directly captured by the two EMA items used in the present study.

A key strength of the present study lies in its use of EMA data to capture the day‐to‐day dynamics of sleep and grief in a clinically relevant population. EMA methods offer greater ecological validity than traditional longitudinal designs, limit biases stemming from retrospective recall, and allow for the modelling of temporal associations at a within‐person level (Shiffman et al. 2008). Further, controlling for autoregressive effects in the lagged models is expected to further increase the robustness of the findings.

Some notable limitations must also be discussed. First, the use of a single‐item sleep measure provides only a narrow view of the potential sleep difficulties that bereaved people may experience. Sleep disturbances that may accompany bereavement are not limited to reports of poor sleep quality, but may also include lack of sleep and nightmares (Lancel et al. 2020; Monk et al. 2008). Indeed, insomnia symptoms (incl. difficulty falling asleep and maintaining sleep, and early morning awakenings), nightmares, and sleep fragmentation appear most consistently linked to PG severity in the broader literature (Lancel et al. 2020). Notably, de Lang et al.'s (2023, 2024) study also examined insomnia symptoms specifically, and found bidirectional associations between insomnia severity and PG levels. It is possible that specific types of sleep disturbance (such as insomnia or nightmares) may show stronger or more reciprocal associations with PG than general sleep quality, as was assessed in our study. Second, our sample size, while powered to detect medium‐sized effects, remains modest for multilevel modelling with random slopes, particularly in the sensitivity analyses. Moreover, the observed effect sizes were small across all models, further reducing the likelihood of detecting such associations of smaller magnitude. Finally, psychopathology commonly co‐occurring with PGD (such as depression or PTSD) may also contribute to sleep disturbances. While we were unable to examine between‐person moderation or subgroup effects (e.g., whether baseline comorbid symptom levels or diagnoses impact the association between sleep and grief) due to power constraints, future research should account for comorbidities when examining sleep–grief links.

In conclusion, this study offers preliminary evidence that disturbed sleep may act as a short‐term risk factor for heightened PG symptoms in daily life. While the present findings warrant replication in larger samples, our results signal to the potential application of sleep‐focused interventions in bereavement care. A pilot study on the efficacy of Cognitive Behavioral Therapy for Insomnia (CBT‐I) for bereaved adults previously found reductions in PG levels in the treatment vs. control group with small‐to‐large effect sizes (depending on follow‐up length), although group differences were not statistically significant (Sveen et al. 2021). These preliminary findings indicate that the applicability of sleep interventions in bereavement care warrants further examination. Both PG and disturbed sleep are associated with adverse physical and mental health outcomes, ranging from poor physiological health markers to increased suicide risk (Bernert 2008; Fernandez‐Mendoza and Vgontzas 2013; Killikelly et al. 2025). Understanding how grief and sleep interact in daily life may help refine interventions both in terms of their timing (e.g., targeting the right symptom at the right time) and content (e.g., whether sleep‐focused treatment may also help reduce PG severity, or vice versa). Overall, our findings underscore the need to move beyond static, one‐time assessments of grief and point towards the clinical importance of capturing the shifting interplay between grief and the factors that influence it in daily life.

## Author Contributions

**Liia M. M. Kivelä:** conceptualization, formal analysis, writing – original draft. **Justina Pociūnaitė‐Ott:** data curation, writing – review and editing. **Lonneke I. M. Lenferink:** conceptualization, data curation, writing – review and editing.

## Funding

This study was supported by the project ‘Toward personalized bereavement care: Examining individual differences in response to grief treatment’ (ID: VI.Veni211G.065) of the research programme (NWO Talent Programme 2021–Veni), which is financed by the Dutch Research Council (NWO) and awarded to Lonneke Lenferink. Justina Pociūnaitė‐Ott is funded by the Trauma Data Institute (TDI).

## Conflicts of Interest

The authors declare no conflicts of interest.

## Supporting information


**Table S1:** Time of day associations between sleep quality and grief.
**Table S2:** Sensitivity analyses of bidirectional associations between grief and sleep quality (*n* = 38).

## Data Availability

The data archive (*Grief‐ID*) (Lenferink and Pociūnaitė‐Ott 2025; Lenferink and Pociūnaitė‐Ott 2025) is available in the Data Archiving and Networked Services (DANS) repository, 10.17026/SS/LNFAXI.

## Endnotes

^1^
As sleep quality during the night was assessed the following morning, in practice, the measurements for Model 1 were gathered at PG symptoms (*t*), sleep quality (*t* + 1) and prior sleep quality (*t*) and for Model 2 at sleep quality (*t*), PG symptoms (*t*) and prior PG symptoms (*t* − 1).

^2^
Post hoc analyses were not pre‐registered.
