# Supplementary material for: Sleepless Longing: Bidirectional Associations Between Sleep Quality and Prolonged Grief in Daily Life After Traumatic Loss
Source: Clin Psychol Psychother. 2026 Feb 12;33(1):e70238. doi: 10.1002/cpp.70238 (PMC12902202; doi:10.1002/cpp.70238)
Supplement: Supplementary file 1 — Table S1: Time of day associations between sleep quality and grief. Table S2: Sensitivity analyses of bidirectional associations between grief and sleep quality (n = 38). [file CPP-33-e70238-s001.docx]

**Sleepless Long – Supplementary Materials 101125**

**Table S1.** *Time of Day Associations between Sleep Quality and Grief*

| **Model** | **B (SE)** | **CI 95%** | **P-VALUE** | **AIC** | **BIC** | **-2 Log-Likelihood** | **Marginal R^2^** | **Conditional R^2^** |
| --- | --- | --- | --- | --- | --- | --- | --- | --- |
| **Model (Sleep + Time of Day > Grief)** |  |  |  | 4477.75 | 4557.37 | 4447.75 | 0.02 | 0.707 |
| Intercept | 2.49 (0.23) | 2.04; 2.94 | < .001 |  |  |  |  |  |
| Sleep | -0.13 (0.04) | -0.21; -0.04 | .003 |  |  |  |  |  |
| Time of Day |  |  |  |  |  |  |  |  |
| Beep 1 | - | - | - |  |  |  |  |  |
| Beep 2 | -0.08 (0.08) | -0.23; 0.07 | .324 |  |  |  |  |  |
| Beep 3 | -0.23 (0.08) | -0.39; -0.07 | .006 |  |  |  |  |  |
| Beep 4 | -0.13 (0.08) | -0.29; 0.03 | .114 |  |  |  |  |  |
| Beep 5 | -0.13 (0.08) | -0.28; 0.02 | .100 |  |  |  |  |  |
| Interaction |  |  |  |  |  |  |  |  |
| Sleep x 1 | - | - | - |  |  |  |  |  |
| Sleep x 2 | 0.18 (0.03) | 0.07; 0.30 | .002 |  |  |  |  |  |
| Sleep x 3 | 0.18 (0.06) | 0.06; 0.30 | .005 |  |  |  |  |  |
| Sleep x 4 | 0.06 (0.06) | -0.07; 0.30 | .371 |  |  |  |  |  |
| Sleep x 5 | 0.08 (0.06) | -0.07; 0.20 | .152 |  |  |  |  |  |
| Grief (t – 1) | 0.19 (0.03) | 0.14; 0.24 | < .001 |  |  |  |  |  |

*Note:* Model includes within-person centred predictors, and an uncentred outcome; beep 1 is the reference category.

**Table S2.** *Sensitivity Analyses of Bidirectional Associations between Grief and Sleep Quality (n = 38)*

| **Model** | **B (SE)** | **CI 95%** | **P-VALUE** | **AIC** | **BIC** | **-2 Log-Likelihood** | **Marginal R^2^** | **Conditional R^2^** |
| --- | --- | --- | --- | --- | --- | --- | --- | --- |
| **Model 1** **(Grief > Sleep)** |  |  |  | 1225.92 | 1252.54 | 1211.92 | 0.001 | 0.424 |
| Intercept | 3.47 (0.20) | 3.15; 3.85 | < .001 |  |  |  |  |  |
| Grief | 0.07 (0.14) | -0.15; 0.35 | .621 |  |  |  |  |  |
| Sleep (t – 1) | 0.03 (0.06) | -0.08; 0.14 | .565 |  |  |  |  |  |
| **Model 2 (Sleep > Grief)** |  |  |  | 978.55 | 1006.60 | 964.55 | 0.005 | 0.830 |
| Intercept | 2.41 (0.23) | 2.03; 2.91 | < .001 |  |  |  |  |  |
| Sleep | -0.05 (0.03) | -0.11; -0.01 | .062 |  |  |  |  |  |
| Grief (t – 1) | 0.11 (0.04) | 0.05; 0.21 | .013 |  |  |  |  |  |

*Note:* Subsample of participants with ≥ 50 % sleep data available. All models include within-person centred predictors, and an uncentred outcome.
